# The endocannabinoid hydrolase FAAH is an allosteric enzyme

**DOI:** 10.1038/s41598-020-59120-1

**Published:** 2020-02-10

**Authors:** Enrico Dainese, Sergio Oddi, Monica Simonetti, Annalaura Sabatucci, Clotilde B. Angelucci, Alice Ballone, Beatrice Dufrusine, Filomena Fezza, Gianni De Fabritiis, Mauro Maccarrone

**Affiliations:** 1 Faculty of Biosciences, and Technology for Food Agriculture and Environment, University of Teramo, Teramo, Italy; 2 European Center for Brain Research (CERC)/Santa Lucia Foundation, Rome, Italy; 3 Faculty of Veterinary Medicine, University of Teramo, Teramo, Italy; 4 Barcelona Biomedical Research Park (PRBB), University of Pompeu Fabra and Icrea, Barcelona, Spain; 5 Department of Experimental Medicine and Surgery, Tor Vergata University of Rome, Rome, Italy; 6 Department of Medicine - Campus Bio-Medico University of Rome, Rome, Italy

**Keywords:** Enzymes, Enzyme mechanisms

## Abstract

Fatty acid amide hydrolase (FAAH) is a membrane-bound homodimeric enzyme that *in vivo* controls content and biological activity of *N*-arachidonoylethanolamine (AEA) and other relevant bioactive lipids termed endocannabinoids. Parallel orientation of FAAH monomers likely allows both subunits to simultaneously recruit and cleave substrates. Here, we show full inhibition of human and rat FAAH by means of enzyme inhibitors used at a homodimer:inhibitor stoichiometric ratio of 1:1, implying that occupation of only one of the two active sites of FAAH is enough to fully block catalysis. Single W445Y substitution in rat FAAH displayed the same activity as the wild-type, but failed to show full inhibition at the homodimer:inhibitor 1:1 ratio. Instead, F432A mutant exhibited reduced specific activity but was fully inhibited at the homodimer:inhibitor 1:1 ratio. Kinetic analysis of AEA hydrolysis by rat FAAH and its F432A mutant demonstrated a Hill coefficient of ~1.6, that instead was ~1.0 in the W445Y mutant. Of note, also human FAAH catalysed an allosteric hydrolysis of AEA, showing a Hill coefficient of ~1.9. Taken together, this study demonstrates an unprecedented allosterism of FAAH, and represents a case of communication between two enzyme subunits seemingly controlled by a single amino acid (W445) at the dimer interface. In the light of extensive attempts and subsequent failures over the last decade to develop effective drugs for human therapy, these findings pave the way to the rationale design of new molecules that, by acting as positive or negative heterotropic effectors of FAAH, may control more efficiently its activity.

## Introduction

Endocannabinoids form a relevant class of lipids that are widespread in tissues, where they exert diverse biological actions^1^. Endocannabinoids have been demonstrated to reduce pain and inflammation, modulate energetic homeostasis and appetite, and have anticancer, anxiolytic, and neuroprotective effects through type-1 and/or type-2 cannabinoid receptors (CB_1_ and CB_2_), as well as via stimulation of transient receptor potential vanilloid channels, peroxisome proliferator-activated receptors, and additional targets^2–4^. Their degradation is due to the activity of several metabolic enzymes, among which FAAH has a prominent role also *in vivo*^5^. In addition, biotransformation of endocannabinoids is catalysed by lipoxygenases (LOXs) or cyclooxygenase-2 (COX-2)^6–8^.

FAAH is a membrane-bound homodimer that is able to hydrolyse AEA and, to a lesser extent, 2-arachidonoylglycerol (2-AG), *N*-oleoylethanolamine^9,10^, *N*-palmitoylethanolamine^11^, and *N-*oleoyltaurine^5,12,13^. The biological activity of AEA, 2-AG and related compounds within the central nervous system^2,3^ and at the periphery has been recently reviewed^6^.

The 3D structures of a truncated form of rat FAAH lacking the N-terminal region^14^ (hereafter referred to as rFAAH), and of its humanized form containing residues of the human active site^15^, have been resolved at high resolution, and some details of the catalytic mechanism of the enzyme have been disclosed^16,17^. In particular, details of substrate selection have been elucidated, leading to the general consensus that structural flexibility of FAAH is a key factor^18,19^.

The crystallographic structure shows that rFAAH is a homodimer, where a transmembrane hydrophobic domain anchors each monomer to the lipid bilayer with a parallel orientation^20^. Indeed, each subunit contains at least two channels, one for the entry of hydrophobic substrates from the membrane side, and the other for the exit of hydrophilic products through a cytosolic gate^14,16^.

Increasing the concentration of endocannabinoids through the inhibition of FAAH has been considered a valuable therapeutic approach to enhance their antinociceptive and anti-inflammatory effects, as well as to protect the nervous system against exogenous insults^18,21–23^. However, extensive attempts over the last decade and subsequent failures to develop clinically effective drugs for human therapy^24–26^ suggest that FAAH is a complex target enzyme. It has been proposed that the parallel orientation of FAAH monomers could in principle allow simultaneous recruitment of two molecules of substrates from the same membrane side^15,16^, but the question of whether a functional communication exists between FAAH monomers leading to an allosteric modulation of the enzyme has remained as yet unanswered.

To interrogate such a possibility, here we analyzed the catalytic properties of rFAAH and two of its mutants, as well as of a full length human FAAH (hFAAH). One of rFAAH mutants (F432A) contains a single substitution known to reside in the active site and proposed to act as a dynamic modulator by *in silico* analysis^27,28^. The other mutant (W445Y) carries a single substitution in a region known to be involved in dimer stabilization^29^. We show that full inhibition of enzyme activity is achieved in rFAAH, its F432A mutant and hFAAH, when only one of the two active sites of the enzyme is occupied by different prototypical inhibitors. Additional kinetic analysis and molecular dynamics simulation further demonstrated that all these FAAHs behave as allosteric enzymes. Instead, the W445Y mutant of rFAAH was not inhibited when half of the active sites were occupied by the same inhibitors, nor did it show an allosteric kinetics. It can be concluded that W445 plays a key role in inter-subunit communication, thus supporting the presence of functional cooperativity. The allosteric control of FAAH implies a fine tuning of its activity within the cell, and opens the possibility to discover and design new non-substrate molecules that, targeting heterotropic allosteric sites of FAAH, can modulate enzyme activity, and potentiate or attenuate the efficacy of FAAH inhibitors.

## Results

### Functional communication between FAAH monomers

A canonical manner to assess an allosteric behaviour of an enzyme is to ascertain the presence of a functional communication between its monomers. To this end, in the case of a homodimer enzymatic activity can be analysed at two homodimer:inhibitor stoichiometric ratios, whereby the inhibitor binds both active sites (1:2 ratio) or one only (1:1 ratio)^30^.

**Figure 1:**
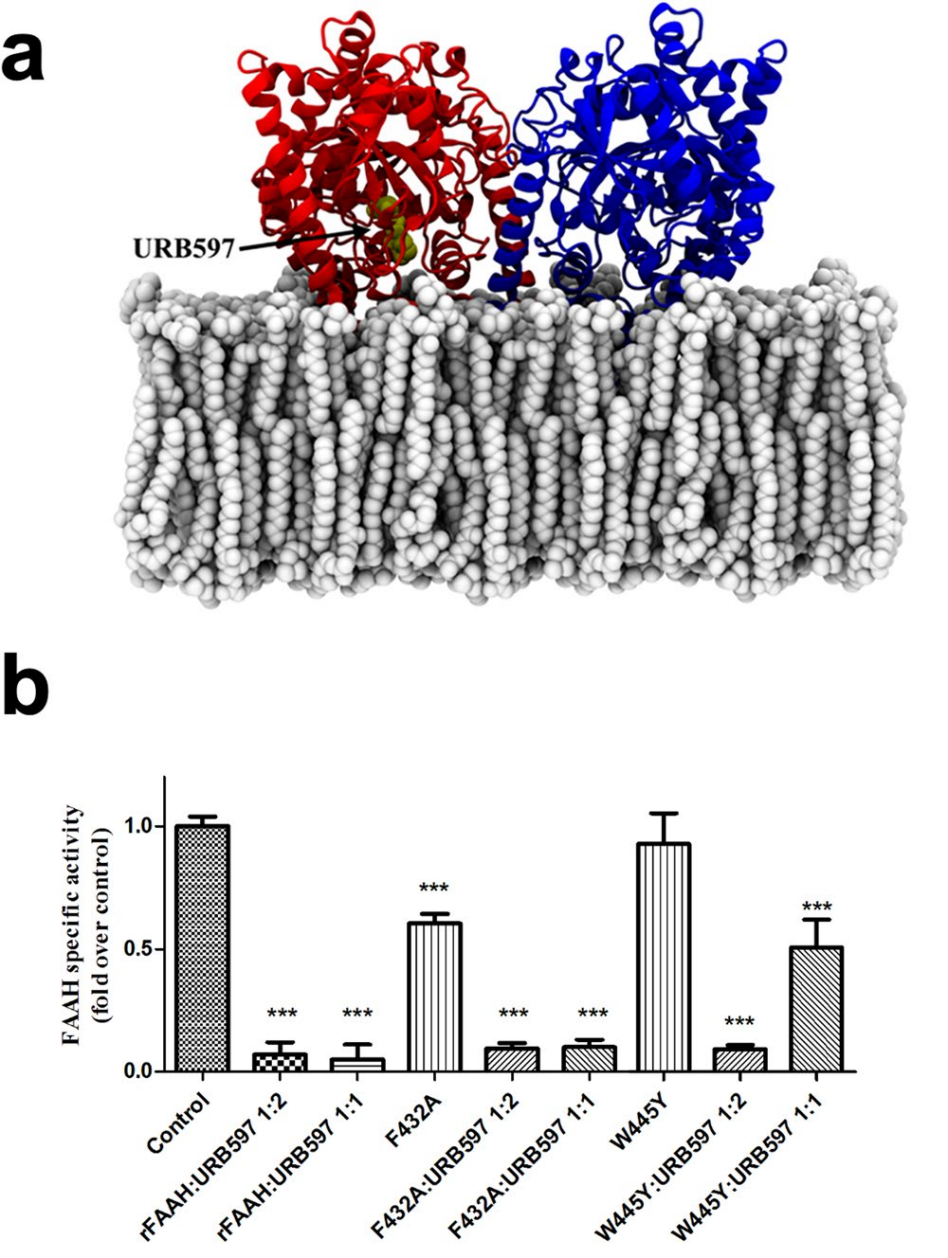
Inhibition of rFAAH and its mutants by URB597 at different molar ratio. (**a**) Schematic model of rFAAH where the URB597 molecule is highlighted in balls and stick within the active site of one subunit of the enzyme; (**b**) from left to right: wild-type rFAAH, rFAAH F432A mutant, and rFAAH W445Y mutant specific activities in the presence of URB597 at 1:2 and 1:1 homodimer:inhibitor molar ratios. ***p < 0.0001.

The specific enzymatic activity of rFAAH was reduced almost completely (>95%) using the selective and irreversible FAAH inhibitor 3′-carbamoyl-[1,1′-biphenyl]-3-yl cyclohexylcarbamate (URB597)^31^ at a homodimer:inhibitor molar ratio of 1:2 (Fig. 1b). Interestingly, the same reduction of rFAAH specific activity was obtained also at a homodimer:URB597 ratio of 1:1 (Fig. 1b). Also other widely used FAAH inhibitors, like the irreversible blockers methoxyarachidonoyl fluorophosphonate (MAFP)^14^, *N*-phenyl-4-(quinolin-3-ylmethyl)piperidine-1-carboxamide (PF-750)^15^ and *N*-pyridin-3-yl-4-[[3-[5-(trifluoromethyl)pyridin-2-yl]oxyphenyl]methyl]piperidine-1-carboxamide (PF-3845)^32^, and the reversible blockers 7-phenyl-1-(5-pyridin-2-yl-1,3-oxazol-2-yl)heptan-1-one (OL-135)^33^ and 1-biphenyl-4-ylethenyl piperidine-1-carboxylate (ST4070)^34^ showed the same effects as URB597 at both molar ratios (Tables 1 and 2). Of note, similar inhibitions of hFAAH were observed with the same blockers at the same homodimer:inhibitor molar ratios used for rFAAH (Tables 1 and 2).

**Table 1:** Residual activity of rFAAH and hFAAH in the presence of prototypical FAAH inhibitors, at a homodimer:inhibitor stoichiometric ratio of 1:2.

	rFAAH (% of specific activity)	hFAAH (% of specific activity)
**CTRL**	100.0 ± 4.2^§^	100 ± 1.0^#^
**URB597**	3.5 ± 0.8***	36 ± 2.0^**^
**MAFP**	2.0 ± 0.5***	0.31 ± 0.01***
**PF-750**	2.9 ± 0.3***	53 ± 11**
**PF-3845**	1.9 ± 0.6***	0.24 ± 0.01***
**OL-135**	4.3 ± 0.9***	62 ± 8**
**ST4070**	4.3 ± 1.3***	68 ±` 4**

^§^rFAAH control specific activity 78.3 ± 9.6 nmol/min per mg of the protein.

^#^hFAAH control specific activity 15.9 ± 2.0 nmol/min per mg of the protein.

**P < 0.01 *versus* hFAAH.

***p < 0.0001 *versus* rFAAH or hFAAH.

Data on rFAAH inhibition by URB597 are from Fig. 1, and are included for the sake of clarity.

**Table 2:** Residual activity of rFAAH and hFAAH in the presence of prototypical FAAH inhibitors, at a homodimer:inhibitor stoichiometric ratio of 1:1.

	rFAAH (% of specific activity)	hFAAH (% of specific activity)
**Control**	100.0 ± 4.2^§^	100 ± 1.0^#^
**URB597**	2.3 ± 1.3***	23.0 ± 3.0**
**MAFP**	3.5 ± 0.4***	5.0 ± 9.0**
**PF-750**	3.6 ± 0.2***	49.0 ± 3.0**
**PF-3845**	3.8 ± 0.2***	2.0 ± 3.0***
**OL-135**	6.8 ± 1.0***	59.0 ± 12.0**
**ST4070**	6.9 ± 0.5***	64.0 ± 17.0**

^§^rFAAH control specific activity 78.3 ± 9.6 nmol/min per mg of protein.

^#^hFAAH control specific activity 15.9 ± 2 nmol/min per mg of the protein.

**p < 0.01 *versus* hFAAH.

***p < 0.0001 *versus* rFAAH or hFAAH.

Data on rFAAH inhibition by URB597 were reported for the sake of clarity, and were taken from Fig. 1.

The amino acid F432 is in the active site of FAAH, where it is involved in activation of the AEA substrate with a pivotal role for substrate hydrolysis^27^. Here, the F432A rFAAH mutant showed a markedly reduced (by half) specific activity compared to the wild-type enzyme (Fig. 1b). Much alike rFAAH, at a 1:1 homodimer:URB597 molar ratio F432A rFAAH mutant was fully inhibited, and so was at 1:2 ratio. These findings demonstrate that, in keeping with a position of F432 far from the monomer-monomer interface^35^, this residue does not contribute to monomer-monomer functional communication.

A relevant rFAAH region proposed to mediate the inter-subunit functional interaction is the area around the evolutionarily conserved residue W445^29^. The latter is indeed a protruding residue that makes the surrounding patch particularly rich in contacts between the two monomers. Thus, we analysed the specific activity of W445Y rFAAH mutant, alone or in the presence of URB597 at 1:2 or 1:1 homodimer:inhibitor stoichiometric ratios. Interestingly, W445Y mutation did not affect rFAAH specific activity *per se*, yet it led to ~50% reduction of enzyme activity at a homodimer:inhibitor ratio of 1:1, and to full inhibition at 1:2 ratio (Fig. 1b). These observations suggest that W445 is involved in the inter-subunit interaction, and that its substitution impairs monomer-monomer functional communication.

### Kinetic properties of wild-type hFAAH, rFAAH, and rFAAH mutants

The same radiometric assay used to assess FAAH inhibition was used to perform kinetic analysis of the wild-type forms of human and rat FAAH (See Table 3 and Supplementary Fig. 1). The calculated Hill coefficient (n_Hill_) values of both rFAAH and hFAAH are suggestive of a positive cooperativity of substrate hydrolysis (Table 3). The binding curve that better fits both rFAAH and hFAAH kinetics is a sigmoid obtained through nonlinear regression analysis using the Hill equation (with values of correlation coefficient R^2^ and χ^2^ of 0.9966 and 579, and of 0.9926 and 154, for rFAAH and hFAAH, respectively), with a K_0.5_ of 12.3 ± 3.1 µM and 8.6 ± 2.7 µM and n_Hill_ of 1.6 ± 0.3 and 1.9 ± 0.3, for rFAAH and hFAAH, respectively (Table 3). Instead, analysis of the same kinetic data through Michaelis-Menten equation yielded a poorer fitting (with values of correlation coefficient R^2^ and χ^2^ of 0.9869 and 1045, and of 0.9521 and 1064, for rFAAH and hFAAH respectively) (Supplementary Fig. 1).

**Table 3:** Kinetic parameters of wild-type hFAAH and rFAAH, and of rFAAH mutants.

	K_0.5_ (µM)	n_Hill_
rFAAH^#^	12.3 ± 3.1	1.6 ± 0.3
hFAAH^#^	8.6 ± 2.7	1.9 ± 0.3
rFAAH	15.7 ± 2.2	1.6 ± 0.2
rFAAH:URB597 (1:0.5)	20.1 ± 3.6	1.6 ± 0.4
rFAAH-F432A	31.0 ± 4.8	1.7 ± 0.4
rFAAH-W445Y	20.0 ± 5.5	0.9 ± 0.2**

Kinetic parameters in all cases were calculated by nonlinear regression analysis of FAAH activity.

^#^These kinetic analyses were done using the radiometric assay (see text and Supplementary Fig. 1).

**p < 0.01 *versus* rFAAH and rFAAH^#^.

We further analyzed rFAAH and its two mutants by means of a fluorogenic assay that is widely used and accepted as a convenient alternative to the radiometric method. Such a fluorogenic assay yielded similar values of the kinetic parameters of the rFAAHs (see Table 3), and was used to perform all subsequent analyses.

**Figure 2:**
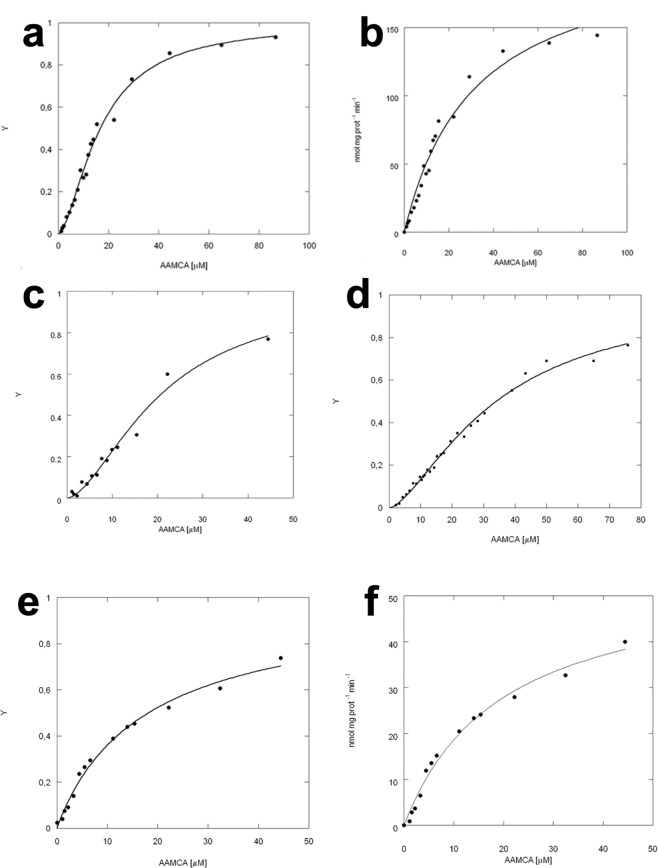
Dependence of rFAAH activity on substrate concentration. (**a)** Dependence of rFAAH activity on substrate concentration, interpolated through the Hill equation; (**b)** rFAAH shows a canonical sigmoidal curve in the presence of increasing concentration of the AAMCA substrate, that is not fitted equally well by non-linear regression through the Michaelis-Menten equation; (**c**) rFAAH in the presence of URB597 at a homodimer:inhibitor 1:0.5 molar ratio shows a sigmoidal behaviour; (**d**) Kinetic analysis of rFAAH F432A mutant indicates that P432 residue is involved in the catalytic activity of the enzyme, but not in the modulation of cooperativity; (**e)** Kinetic analysis of rFAAH W445Y mutant interpolated through the Hill formalism; (**f)** Kinetic analysis of rFAAH W445Y mutant shows loss of the sigmoidal behaviour, leading to a canonical hyperbolic Michaelis-Menten enzyme without any cooperativity.

Even by using the fluorogenic assay, we found that the isotherm that better described rFAAH kinetics had a sigmoidal shape (Fig. 2a), that could be fitted by Hill equation (with correlation coefficient R^2^ and χ^2^ values of 0.9952 and 412, respectively) with a K_0.5_ of 15.7 ± 2.2 µM and a n_Hill_ of 1.6 ± 0.2 (Table 3). Instead, analysis of the same kinetic data through Michaelis-Menten equation yielded a poorer fitting (with values of correlation coefficient R^2^ and χ^2^ of 0.9869 and 1098, respectively) (Fig. 2b). The calculated n_Hill_ values of both rFAAH and hFAAH are suggestive of a positive cooperativity of substrate hydrolysis (Table 3).

At a homodimer:inhibitor ratio of 1.0:0.5, rFAAH showed a sigmoidal substrate dependence (Fig. 2c) with an increased K_0.5_ value and the same n_Hill_ value of those of rFAAH alone (Table 3). These results demonstrate that at a 1.0:0.5 stoichiometry, the amount of inhibitor is not enough to saturate at least one of the two active sites; thus, under these experimental conditions, the higher value of K_0.5_ compared to that calculated in the absence of inhibitor seems to reflect a reduced amount of inhibitor-free enzyme (Table 3).

In addition, kinetic analysis of F432A rFAAH showed a cooperative behaviour (Fig. 2d) with an increased value of K_0.5_ and the same n_Hill_ values as wild-type rFAAH (see Table 2). Instead, kinetic analysis of W445Y rFAAH showed a loss of cooperativity (Fig. 2e), with a sigmoidal shape and R^2^ and χ^2^ values of 0.9721 and 1144, respectively. Of note, the kinetic data of the latter mutant were better fitted by Michaelis-Menten equation, with R^2^ and χ^2^ values of 0.9910 and 387, respectively (Fig. 2f), and n_Hill_ value ~1.0 (Table 3). These findings strongly indicate that W445Y substitution completely impairs rFAAH cooperativity.

### Molecular dynamics simulations

**Figure 3:**
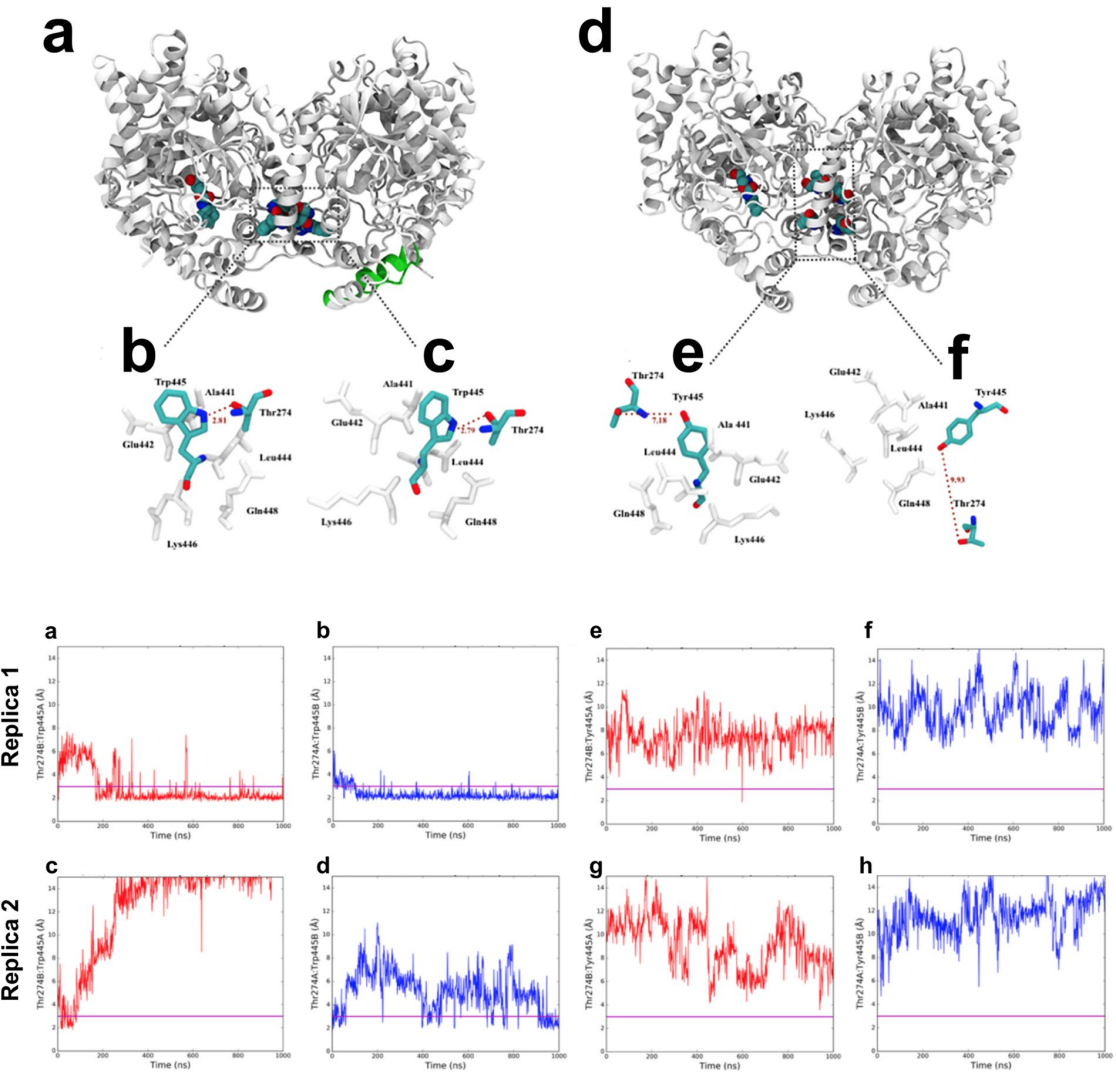
Molecular dynamics of rFAAH and its W445Y mutant. Upper Panel: Last frame snaphots of MD simulations of the URB597/rFAAH (**a**) and the URB597/W445Y-rFAAH complexes (**d**), where the URB597 molecule is covalently bound in the active site of one monomer only (monomer A). Schematic representation of most frequent W445:T274 interactions (**b**,**c**) and Y445:T274 interactions (**e**,**f**) over the simulation is reported; related residues are also depicted in the dotted squares (**a**,**d**). Distances between residues are indicated with red broken lines (**b**,**c**,**e**,**f**). Lower Panel: The HB plots URB/rFAAH complex (**a**–**d**), where distances (expressed in Å) between the OH group of T274 and the W445’s sidechain nitrogen over all the trajectory length, are shown. These simulations indicate that the T274 of monomer A of wild type rFAAH establishes a hydrogen bond with the W445 of monomer B (and *viceversa*) in 3 cases of the 4 analyzed trajectories length. The HB Plots of URB/W445Y-rFAAH complex (**e**–**h**) show that in the rFAAH mutant the distances between the OH group of T274 and Y445’s sidechain hydroxyl in both monomers and all replicas (**e**–**h**) are not compatible with the formation of a hydrogen bond. These MD simulations suggest that the hydrogen bond is more likely to form in the wild-type rFAAH. A line y = 3 highlights peaks over 3 Å for each of the plot.

In order to have indications on the possible molecular mechanism behind the allosteric behaviour of rFAAH, we performed high-throughput molecular dynamics (MD) simulations on two systems: URB597 bound to wild-type rFAAH (URB597/rFAAH complex) and to its W445Y mutant (URB597/W445Y-rFAAH complex). Inhibition of rFAAH by URB597 occurs through cleavage of the carbamate bond of the inhibitor^36^, thus we modelled only the cyclohexane aminocarboxylic acid moiety of URB597 in the active site of one monomer (referred to as monomer A) of the enzyme (Fig. 3).

In the URB597/rFAAH complex we found a direct interaction between the two monomers that may explain the allosteric nature of the dimer. Indeed, in monomer A the loop comprising residues 266–275 contains an OH group (Oγ) of T274 that forms a hydrogen bond with side chain nitrogen of W445 in monomer B, and *viceversa*. The distance between these residues was often 2–3 Å over the entire trajectories (Fig. 3 **upper panel a,b; lower panel, a-d**). Instead, we failed to observe a similar interaction for URB597/W445Y-rFAAH, because distances between Y445 and T274 were >6 Å in all trajectories (Fig. 3 **upper panel, d,f; lower panel, a-d**).

## Discussion

Our study demonstrates for the first time that FAAH — the main hydrolase cleaving AEA and related compounds — is an allosteric enzyme. There are several lines of evidence in support of this claim: (*i*) FAAH is composed of two identical subunits symmetrically arranged^14^; (*ii*) the wild-type enzyme is fully inhibited when only one subunit of the dimer is bound to one inhibitor molecule, but not in the W445Y mutant, where a substitution is made in the inter-subunit region (Fig. 1); (*iii*) kinetic analysis shows a sigmoidal dependence of FAAH-catalyzed reaction on AEA concentration, while the W445Y mutant shows a hyperbolic curve **(**Fig. 2**)**; (*iv*) molecular dynamics simulations suggest that the mechanism of cooperativity of FAAH involves W445, which could possibly transmit the conformational change from one subunit to the other, thus controlling the active site accessibility.

Quaternary structure is a necessary, but not sufficient, condition for a protein to display allosteric properties. X-ray crystallographic studies^14,15^ showed that FAAH protein forms high molecular weight oligomers, but suggested the homodimer as the biological structure of both rFAAH and hFAAH. In line with this, we also found an oligomeric organization of FAAH in solution and demonstrated that FAAH oligomers are present only in small amounts (5–8%); once dissociated into homodimers (also due to stabilization by biological membranes), the latter showed the same kinetic properties as the oligomers of FAAH^20^. These data, together with the cooperative behaviour of FAAH reported here, indicate the FAAH homodimer as the allosteric functional unit of the enzyme.

To ascertain the possible allosteric communication between the two monomers of FAAH, we assayed the effects on its catalytic activity of selective inhibitors at a stoichiometric ratio of 1:1 with respect to the dimeric form, an approach successfully employed to demonstrate allosterism of COX-1^23^ and COX-2^30,37^, two enzymes with a structural topology similar to that of FAAH. Inhibition of the catalytic activity of both rFAAH and hFAAH is comparable at both homodimer:inhibitor stoichiometric ratios of 1:1 and 1:2 (Tables 1 and 2). This finding demonstrates that occupancy of one active site with the inhibitor molecule is able to impair the catalytic activity also in the other (unoccupied) active site, and strongly supports the presence of a functional cross-talk between the two subunits of FAAH.

It is noteworthy that both irreversible inhibitors (like URB597, MAFP, PF-750, PF-385), and reversible blockers (like OL-135 and ST4070) yielded the same results, suggesting that the mechanism of inhibition was not relevant. Incidentally, the different potencies of the same inhibitor on rFAAH compared to hFAAH are not unprecedented^17,30^, and are likely due to structural differences between the two enzymes^38^.

Kinetic analyses of rFAAH and hFAAH strongly support the allosteric nature of FAAH suggested by the inhibition studies. In particular, the values of Hill coefficients (Fig. 2a,b and Table 3) imply that FAAH has homotropic substrate cooperativity. Functional studies of site-directed mutant enzymes further demonstrated that F432A mutant retained homotropic substrate cooperativity, while the W445Y mutant completely lost cooperativity, confirming that the mechanism of FAAH allosterism involves W445 but not F432 (Fig. 2e,f).

In order to get additional information on the experimentally demonstrated FAAH allosterism, we performed microsecond MD simulations of rFAAH with only one URB597 molecule bound at one of the two monomers. Notably, MD simulations revealed the presence of two specific hydrogen bonds in the rFAAH homodimer (Fig. 3). In particular, for the URB597/wt-rFAAH complex, the *in silico* analysis suggests the presence of a stable interaction between residues W445 and T274 of both monomers.

Our MD approach showed that substitution of W445 with a tyrosine residue was sufficient to prevent the formation of the two hydrogen bonds in W445Y mutant. Unfortunately, our simulations did not allow to decipher the underlying allosteric mechanism. However, it should be noted that the position of the conserved residue W445 — fully buried within the FAAH core and located at the interface between the two subunits — makes this residue particularly suitable to propagate conformational changes from one active site of the enzyme to the other. Concerning T274, this was suggested by MD simulation as a possible interacting residue with W445, *via* a quite stable hydrogen bond in rFAAH. However, T274 is only conserved both in rFAAH and mouse FAAH, while in the human enzyme, at the same position, there is a glutamate (E274), a residue that could still form a hydrogen bond with W445. Thus, it can be speculated that dynamic interaction among W445 of one monomer and polar residues located in the 266–275 loop region of the other monomer (as the T274 here suggested by MD for rFAAH) could allosterically control the structure of the enzyme, thus regulating substrate accessibility to the catalytic site of FAAH. However, these mechanicistic aspects deserve to be analyzed with a more dedicated study.

Allosteric enzymes are known to be placed at key points of metabolic pathways, in order to finely regulate their fluxes according to the specific state of the cell. In line with this, it is tempting to speculate that an allosteric FAAH is suitable to finely tune intensity and duration of signalling by AEA, as well as by other bioactive fatty acid amides that are enzyme substrates, such as *N*-oleoylethanolamine, *N*-palmitoylethanolamine and *N*-oleoyltaurine^9,10^. At any rate, an allosteric regulation of FAAH emphasizes the biological relevance of this enzyme as a key controller of endocannabinoid signalling. In this context, accumulated evidence demonstrates a complex interplay among distinct FAAH substrates, and it is likely that an overall inhibition of enzyme activity can alter the spatiotemporal interactions among these bioactive lipids, and transduction pathways thereof^39^. For instance, promising results have been obtained by dissociating FAAH-catalysed hydrolysis of *N*-acylethanolamines *versus N*-acyltaurines through structure-guided design of a point mutant in the cytoplasmic access tunnel of the enzyme^19^. A similar approach has also been successfully used to design “substrate-selective” COX-2 inhibitors that prevent endocannabinoid inactivation without affecting prostaglandin generation from arachidonic acid^8,40^. Therefore, it is possible that also substrate preference of FAAH could change in the presence of suitable inhibitors (at a 1:1 ratio), with a clear impact on cell signalling under pathophysiological conditions.

On a final note, pharmacological and genetic augmentation of endogenous fatty acid amides is known to produce pleiotropic actions, including analgesic^22^, anxiolytic^34^, anti-inflammatory^21^, and anti-depressant effects^41^. Unsurprisingly, in the last few years FAAH has been the target of several drug development programmes, aimed at treating a broad range of human pathological conditions^24^. Our present data suggest that allosteric drug development could be a novel pharmacological strategy for controlling in a more selective and efficient manner the “catalytic promiscuity” of FAAH. Indeed, allosteric sites are structurally much less conserved than catalytic sites, thus supporting the development of drugs — positive or negative heterotropic modulators — with much greater selectivity. In this context two phenoxyacyl-ethanolamides, 3-*n*-pentadecylphenolethanolamide and cardanolethanolamide, have been described as FAAH activators^42^, and it can be speculated that these non-hydrolysable analogues of *N*-acylethanolamines may stimulate hydrolysis of AEA and congeners by acting as positive allosteric modulators of FAAH.

In conclusion, we provide unprecedented evidence for an allosteric regulation of FAAH, providing unexplored opportunities to design and develop novel therapeutic drugs targeted to regulatory site(s) of this major AEA-inactivating enzyme.

## Methods

### Reagents and enzymes

All chemicals were of the purest analytical grade. AEA, arachidonic acid, ethanolamine, and Protease Inhibitor Cocktail were from Sigma Chemical Co. (St. Luis, MO USA). IPTG was purchased at Promega Corporation (Wisconsin, USA). The *E. coli* BL21(DE3)pLysS competent cells were purchased from Merck KGaA (Darmstadt, Germany). Threalose was from Cargill (Cargill Incorporated, Minneapolis, USA). [^3^H]-AEA was from Larodan (LARODAN Fine Chemicals AB, Malmö, Sweden). AAMCA was from BIOMOL (BIOMOL International, USA). The Talon resin was from Clontech (California, USA). All other chemicals were purchased from Sigma Chemical Co. (Milan, Italy), unless stated otherwise.

ΔTM rFAAH was purified as reported^20^. In the F432A rFAAH mutant the protein codon TTT at position 1355 of cDNA was mutated into GCT resulting in the substitution of the phenylalanine in position 432 of the protein sequence with alanine. In the W445Y rFAAH mutant the codon TGG was mutated in position 1394 of the cDNA into TAC obtaining a consequent substitution of tryptophan in position 445 of the protein sequence with tyrosine. The catalytically active F432A and W445Y rFAAH mutants were expressed in *Escherichia coli* with a hexahistidine tag using the prokaryotic expression vector pTrcHisA, and were extracted from cell lysates according to the protocol of Patricelli *et al*.^35^ x with modifications. Briefly, the proteins were expressed in the competent cells according to the manufacturer’s guidelines. To improve the protein recovery, cell pellets were subjected to mechanical rupture using the following lysis buffer: 0.5 M Tris/HCl, pH 7.5, 0.1 M NaCl, 0.5% (w/v) CHAPS, 10% (w/v) trehalose. Lysozyme at a final concentration of 1 mg/mL and a 10 μl/mL Protease Inhibitor Cocktail were added to the lysis buffer. The resulting lysate was then sonicated with a tip sonicator (Bandelin, Berlin Germany) with three 10 s pulses (50% power), and then centrifuged at 10,000 × *g* for 30 min at 4 °C. Subsequently, the proteins were purified by metal affinity chromatography with the Talon cobalt affinity resin (Clontech). The column was pre-equilibrated with 10 column volumes of 0.5 M Tris/HCl, pH 7.5, 0.1 M NaCl, 10% (w/v) trehalose (column buffer) containing 10 mM imidazole and washed twice with column buffer containing 15 mM and 20 mM imidazole, respectively, for the removal of unspecifically bound proteins. Each FAAH sample was completely eluted after the addition of 3 column volumes of elution buffer (column buffer containing 200 mM imidazole). The purified enzymes were dialyzed overnight with the column buffer to completely remove imidazole and stored at −20 °C until use.

### FAAH activity assay

Enzymatic activity of rFAAH, its mutants and hFAAH was assayed by measuring the release of [^3^H]-ethanolammine from [^3^H]-AEA (60 Ci/mmol) (Larodan Fine Chemicals AB, Malmo Sweden), using liquid scintillation counting according to the method of Gattinoni and coworkers^43^. In substrate-dependence experiments, that were performed including several data points in the low substrate concentration range, enzymatic activity of rFAAH and its mutants was assayed by means of the fluorescent-based method by Ramarao and colleagues^44^, by measuring the release of arachidonic acid and of the highly fluorescent 7-ammino**-**4**-**methyl coumarin (AMC) product from the non-fluorescent arachidonyl 7-ammino**-**4**-**methyl coumarin amide (AAMCA) substrate of FAAH. In preliminary experiments both methods (radiometric and fluorimetric) were found to yield the same catalytic constants, that were in keeping with literature data^15,34,45^. Kinetic analysis were done both in the presence and in the absence of 1-palmitoyl-2-oleyl-sn-glycero-3-phosphocholine (POPC) large unilamellar vesicles prepared as described previously^46^.

Protein concentration was determined spectrophotometrically, assuming a 0.1% extinction coefficient at 280 nm of ε = 1.11, as calculated from the primary structure using the ‘prot param’ tool in the expasy proteomic server (www.expasy.org). In all FAAH enzymatic assays carried out at a 1:1 homodimer:inhibitor stoichiometric ratio, 40 nm of the FAAH homodimer (*i.e*., with two active sites) and 40 nm of all the evaluated inhibitors were used. For the 1:2 homodimer:inhibitor stoichiometry 40 nm of FAAH and 80 nm of inhibitor concentration were used. All enzymatic assays were carried out at 37 °C using a pre-incubation time of 20 minutes of all the evaluated inhibitors with the different FAAHs before adding the substrate (final volume of 500 μL).

Kinetic parameters of the FAAH-catalyzed reaction were calculated from dose-dependence curves (0–100 μM substrate range) through nonlinear regression analysis using the software Kaleidagraph (Synergy Software). Allosteric behaviour was analysed by non-linear regression analysis of kinetic data using the Hill equation $Y=1/\{1+{({K}_{0.5}/[S])}^{n}\}$ and the MWC formalism^47,48^.

### Molecular dynamics

As previously done^20^, molecular dynamics studies were carried out in the presence of a POPC membrane. To better parallel these *in silico* data, we also evaluated the kinetic properties of FAAH in the presence and in the absence of POPC membranes. In agreement with previous data^20^, no differences were found in enzyme activity under the two experimental conditions (data not shown). Two replicas for each rFAAH-membrane system were prepared for molecular dynamics simulations. Membrane position was taken from the OPM database using the HTMD software and run using ACEMD^49^. Input coordinates of the systems were based on PDB code 1MT5, from where the inhibitor methyl arachidonoyl fluorophosphonate (MAFP), covalently bond to the catalytic nucleophile S241, was removed. The crystal structure of the FAAH-URB597 (PDB code 3LJ7) complex was used for superimposition in our URB597-bound models. The all-atom CHARMM36 force field was used for the protein, lipid and water atoms. With an identical setup also the W445Y mutation was prepared. For each complex, we performed two independent simulations of 1 μs, giving a final aggregate of 4 μs length. More details about the set-up of the inhibitor covalently bond to the protein are reported in the Supplementary Information.

### Statistical analysis

Data reported in this paper are the mean (±S.D.) of at least three independent determinations, each performed in triplicate. Statistical analysis was performed by the non-parametric Mann-Whitney U test, analyzing experimental data by means of the Prism 5 program (GraphPAD Software for Science, San Diego, CA).

## Supplementary information

Supplementary information.
