# Author Correction: The endocannabinoid hydrolase FAAH is an allosteric enzyme

**DOI:** 10.1038/s41598-020-62514-w

**Published:** 2020-03-31

**Authors:** Enrico Dainese, Sergio Oddi, Monica Simonetti, Annalaura Sabatucci, Clotilde B. Angelucci, Alice Ballone, Beatrice Dufrusine, Filomena Fezza, Gianni De Fabritiis, Mauro Maccarrone

**Affiliations:** 1 Faculty of Biosciences, and Technology for Food Agriculture and Environment, University of Teramo, Teramo, Italy; 2 European Center for Brain Research (CERC)/Santa Lucia Foundation, Rome, Italy; 3 Faculty of Veterinary Medicine, University of Teramo, Teramo, Italy; 4 Barcelona Biomedical Research Park (PRBB), University of Pompeu Fabra and Icrea, Barcelona, Spain; 5 Department of Experimental Medicine and Surgery, Tor Vergata University of Rome, Rome, Italy; 6 Department of Medicine - Campus Bio-Medico University of Rome, Rome, Italy

Correction to: *Scientific Reports* 10.1038/s41598-020-59120-1, published online 10 February 2020

In the original version of this Article, Alice Ballone was incorrectly affiliated with ‘Faculty of Biosciences, and Technology for Food Agriculture and Environment, University of Teramo, Teramo, Italy’. The correct affiliation is listed below.

Barcelona Biomedical Research Park (PRBB), University of Pompeu Fabra and Icrea, Barcelona, Spain.

This error has now been corrected in the PDF and HTML versions of the Article.

