# Supplementary material for: The endocannabinoid hydrolase FAAH is an allosteric enzyme
Source: Sci Rep. 2020 Feb 10;10:2292. doi: 10.1038/s41598-020-59120-1 (PMC7010751; doi:10.1038/s41598-020-59120-1)
Supplement: Supplementary file 1 — Supplementary information. [file 41598_2020_59120_MOESM1_ESM.docx]

**Supplementary Information**

**The endocannabinoid hydrolase FAAH is an allosteric enzyme**

Enrico Dainese^1,2*§^, Sergio Oddi ^2,3§^, Monica Simonetti^1^, Annalaura Sabatucci^1^, Clotilde B. Angelucci^3^, Alice Ballone^4^, Beatrice Dufrusine^1^, Filomena Fezza^5^, Gianni De Fabritiis^4^, Mauro Maccarrone^2,6*^

*^1^Faculty of Biosciences, and Technology for Food Agriculture and Environment, University of Teramo, Teramo, Italy, ^2^European Center for Brain Research (CERC)/Santa Lucia Foundation, Rome, Italy, ^3^Faculty of Veterinary Medicine, University of Teramo, Italy,* *^4^Barcelona Biomedical Research Park (PRBB), University of Pompeu Fabra, Barcelona, Spain,* ^5^*Department of Experimental Medicine and Surgery, Tor Vergata University of Rome, Rome, Italy,* ^6^*Department of Medicine - Campus Bio-Medico University of Rome, Rome, Italy.*

*Short title*: Allosteric FAAH

^§^Equally first authors

**Corresponding authors*: e-mail: edainese@unite.it, tel. and fax +39 0861 266876; m.maccarrone@unicampus.it, tel. and fax: +39 06 2254 19169.

**Legends to Supplementary Figure**

**Supplementary Figure 1. Dependence of rFAAH and hFAAH activities on substrate concentration.** Both rFAAH (A) and hFAAH (B) show a substrate dependence that is better fitted by the Hill equation than by non-linear regression through the Michaelis-Menten equation (see text for further details).

**Supplementary Figure 1**

**
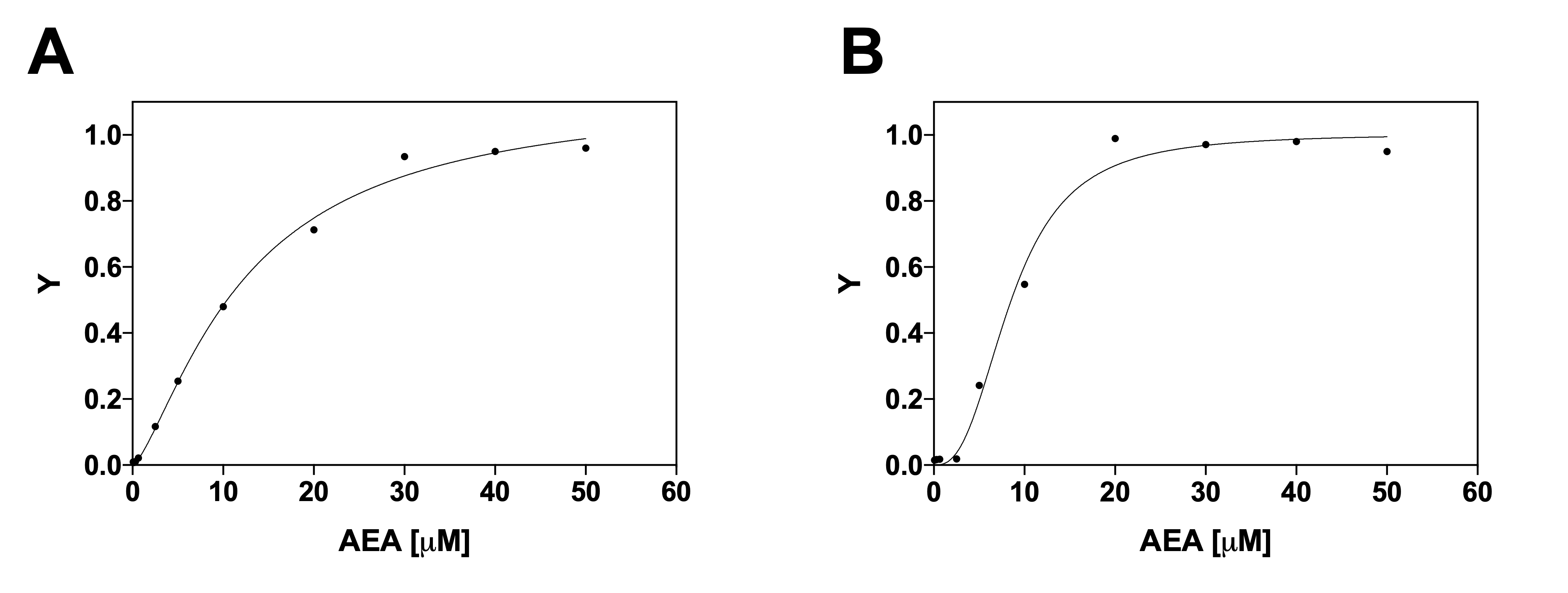
**

**Supplementary Methods**

***Molecular dynamics***

All systems were prepared using HTMD ^1^, embedded in a POPC membrane, solvated in 0.15 M NaCl and kept them electrically neutral. Each system was minimized and relaxed under NPT conditions for 24 ns at 1 atm and 300 K using a time-step of 4 ps, rigid bonds, cut-off of 9 Å and PME for long range electrostatics using the software ACEMD^2^. Production simulations were run in an in-house workstation equipped with graphical processing units (GPUs), having a time-step of 4 fs at 1 atm and 300 K using a Langevin thermostat with damping of 0.1 ps. Each replica was run for 1 μs giving a total ensemble of simulation time of 4 μs. During the production runs, a flat-bottom restraining potential with a size of ~15 Å x ~35 Å x ~20 Å was applied to each monomer’s center of mass. The flat-bottom, without affecting the physical properties of FAAH-membrane system, prevented the rotation of the protein during the simulations. The force constant used for the flat-bottom restraint was 5 kcal/mol/A^2^.

*Building URB597 covalently bound to FAAH*

We used Corina molecular editor software (available at http://www.molecular-networks.com/online_demos/corina_demo_interactive) to obtain the 3D-structure of the non-standard residue formed by the inhibitor URB597 covalently bonded to the catalytic nucleophile S241 of FAAH. To interconvert the file format MOL obtained by Corina to MOL2 file, we used HTMD in order to parameterize the molecule by a derivative of the GAAMP tool included in HTMD. To define a RESI in the new topology file, coordinates of the S241 were taken from the CHARMM22star topology.

***References***

1. Doerr, S., Harvey, M. J., Noé, F. & De Fabritiis, G. HTMD: High-Throughput Molecular Dynamics for Molecular Discovery. *J. Chem. Theory Comput.* **12**, 1845–52 (2016).

2. Harvey, M. J., Giupponi, G. & Fabritiis, G. De. ACEMD: Accelerating Biomolecular Dynamics in the Microsecond Time Scale. *J. Chem. Theory Comput.* **5**, 1632–9 (2009).
